# Improving the Reliability of Peer Review Without a Gold Standard

**DOI:** 10.1007/s10278-024-00971-9

**Published:** 2024-02-05

**Authors:** Tarmo Äijö, Daniel Elgort, Murray Becker, Richard Herzog, Richard K. J. Brown, Benjamin L. Odry, Ron Vianu

**Affiliations:** 1 Covera Health, New York, NY USA; 2 Present Address: Aster Insights, Tampa, FL USA; 3 Rutgers Robert Wood Johnson Medical School, New Brunswick, NJ USA; 4 Department of Radiology, University of Michigan (Michigan Medicine), Ann Arbor, MI USA

**Keywords:** Reliability, Peer review, Gold standard

## Abstract

Peer review plays a crucial role in accreditation and credentialing processes as it can identify outliers and foster a peer learning approach, facilitating error analysis and knowledge sharing. However, traditional peer review methods may fall short in effectively addressing the interpretive variability among reviewing and primary reading radiologists, hindering scalability and effectiveness. Reducing this variability is key to enhancing the reliability of results and instilling confidence in the review process. In this paper, we propose a novel statistical approach called “Bayesian Inter-Reviewer Agreement Rate” (BIRAR) that integrates radiologist variability. By doing so, BIRAR aims to enhance the accuracy and consistency of peer review assessments, providing physicians involved in quality improvement and peer learning programs with valuable and reliable insights. A computer simulation was designed to assign predefined interpretive error rates to hypothetical interpreting and peer-reviewing radiologists. The Monte Carlo simulation then sampled (100 samples per experiment) the data that would be generated by peer reviews. The performances of BIRAR and four other peer review methods for measuring interpretive error rates were then evaluated, including a method that uses a gold standard diagnosis. Application of the BIRAR method resulted in 93% and 79% higher relative accuracy and 43% and 66% lower relative variability, compared to “Single/Standard” and “Majority Panel” peer review methods, respectively. Accuracy was defined by the median difference of Monte Carlo simulations between measured and pre-defined “actual” interpretive error rates. Variability was defined by the 95% CI around the median difference of Monte Carlo simulations between measured and pre-defined “actual” interpretive error rates. BIRAR is a practical and scalable peer review method that produces more accurate and less variable assessments of interpretive quality by accounting for variability within the group’s radiologists, implicitly applying a standard derived from the level of consensus within the group across various types of interpretive findings.

**Supplementary Information:**

The online version contains supplementary material available at 10.1007/s10278-024-00971-9.

## Introduction

In radiology, quality improvement (QI) programs are key to attaining the goals of both improving patient outcome and increasing the value of care [1, 2]. To achieve these goals, one key element of a QI program is the need to evaluate a radiologist’s interpretive performance, which requires a program of continuous assessment and feedback throughout the radiologist’s career [3]. Quantitative metrics, such as “interpretive error rate” seem well suited for such continuous training because they can be tracked longitudinally and be used to generate feedback [4]. However, deriving accurate, reliable, and scalable error rates has proven challenging, and radiologists often lack confidence in these measurements [5, 6]. The shift from peer review to peer learning has, in part, been driven by this lack of confidence in the peer review process [7]. Thus, one challenge when constructing a quality program that includes ongoing professional feedback is defining a reliable methodology for measuring radiologist performance and demonstrating the method’s efficacy.

Comparison of a radiologist’s interpretation with a follow-up gold standard test is one method for calculating error rates. For example, the rate of missed interval cancers at screening mammography can be estimated by comparing breast cancer biopsy results with prior mammographic imaging studies and interpretive reports [8]. However, this method cannot be scaled broadly across radiology because similar gold standards are not available for many imaging exams. Moreover, when calculating error rates, it is important to consider the clinical significance of the errors and the possibility that the gold standard test is positive even though the pathology is not visible in images.

Fault detection (e.g., detection of misdiagnosis) is a desired property of many mission-critical systems. Peer review in radiology has some similarities to fault detection approaches used in, e.g., computing systems where computation is performed multiple times using spare capacity, and the obtained results are compared to increase the “trustability” of the system [9–11] or using redundant sensors and decision by majority [12]. As a practical alternative to gold standards, QI programs may use subjective secondary image interpretation or multiple image interpretations (peer review) to flag discrepancies in the findings reported on the initial interpretation and use these discrepancies as proxies for interpretive errors [13]. A concern with this approach is that reviewers themselves introduce subjectivity and errors and vary in their sensitivity to “calling” errors or definition of error. Second, performing enough peer reviews to enable the calculation of statistically valid interpretive error rates can require substantial time and resources, which can be compounded when incorporating methodology to address the problem of inter-reviewer variability using multiple image interpretations per study.

In this paper, a novel approach for using peer review to measure interpretive error rates is presented called the “Bayesian Inter-Reviewer Agreement Rate” (BIRAR) method, which recognizes that reviewers will be imperfect but does not require all exams sampled by the QI program to be peer-reviewed multiple times in order to reach a consensus-based gold standard. Due to the modeling of intrinsic subjectivity in peer review data, BIRAR has the capability to consider interpretive variability leading to more robust conclusions from data. The BIRAR formulation is inspired by the previous work on Bayesian models of annotation [14]. This paper compares the accuracy and reliability of the BIRAR method with alternate methods using peer reviews to assess interpretive error rates, including a method that uses gold standard diagnosis. In addition, this paper also investigates the impact of reviewer error rates and overall QI case volume on the relative efficacy of these methods.

## Materials and Methods

### Summary of Study Design

This study compares alternative methods for using peer reviews of imaging studies to measure the interpretive error rates of a population of radiologists. The term “interpretive error” is defined as any instance in which the original reading radiologist or the peer-reviewing radiologist assesses the presence or grading of the patient’s pathology to be different from the “true state” of the pathology that is depicted in the radiological images.

**Fig. 1:**
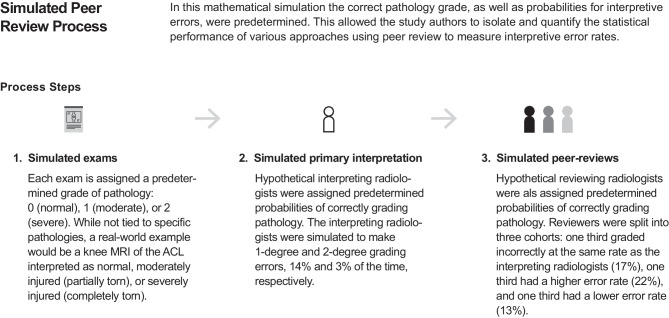
Overview of the computer-simulated peer review process that is used in this study to compare the performance of various approaches for using peer reviews to measure interpretive error rates

There are many reasons why the “true state” of the patient’s pathology may be indeterminate or unknown (e.g., pathology not clearly visible in images, borderline severity, or variable nomenclature), which makes it difficult or impossible to categorize some reported diagnostic findings as interpretive errors. However, this study uses computer-simulated peer review data, where a software simulation generates counts of patient exams and peer reviews that contain interpretive errors, given that the radiologists performing the initial interpretations and peer reviews of these exams had predefined probabilities of making interpretive errors of specific types (Fig. 1).

Using the simulation also enables an explicit comparison to be made between the interpretive error rates that are assigned to each evaluated radiologist and the error rate that is then calculated using the peer review–based methods. The interpretive error rates calculated by the peer review–based measurement methods will deviate from the evaluated radiologists’ true error rates (predefined by the simulation) for two reasons: (1) the peer reviewers are also assigned interpretive error rates, and (2) the peer reviews only cover a finite sample of exams from each evaluated radiologist that are selected for review.

**Fig. 2:**
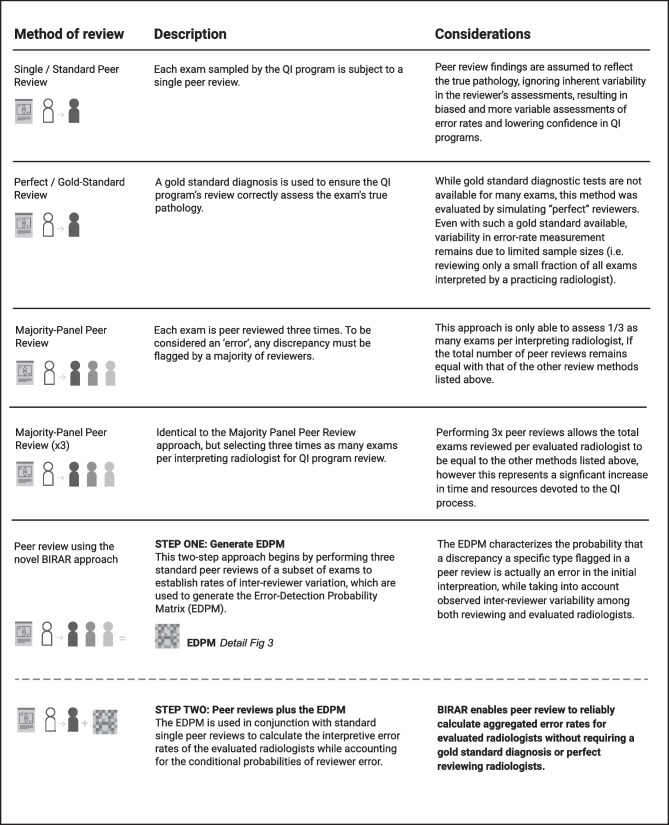
Five approaches evaluated in this study for calculating the interpretive error rates of 100 radiologists using simulated peer review data

The five peer review approaches evaluated in this study to calculate interpretive error rates using simulated peer review data are depicted in Fig. 2.

To evaluate each of the five approaches described in Fig. 2, a set of simulated peer reviews was used to calculate the interpretive error rates for 100 hypothetical radiologists. For the “Single / Standard Peer Review” and “Perfect / Gold-Standard Review” approaches, scenarios were simulated in which 90 exams were sampled for peer review from each evaluated radiologist, requiring a total of 9000 aggregate peer reviews. For the “Majority Panel Peer Review” approach, only 30 exams per evaluated radiologist were subjected to simulated triple peer review in order to align with the aggregate total of 9000 peer reviews used to assess all 100 radiologists. For the “Majority Panel Peer Review × 3” approach, 27,000 peer reviews were simulated (90 exams selected for review from each of the 100 radiologists, each peer reviewed three times). For the BIRAR approach, in “Step One,” a triple peer review of 300 exams was simulated to generate the EDPM, and then in “Step Two,” 81 single peer reviews per evaluated radiologist were simulated in order to align with the aggregate total of 9000 peer reviews used to assess all 100 radiologists.

### Calibration of Radiologist Error Rates

For the purposes of this simulation study, the hypothetical diagnostic imaging exams were defined to be ones in which a radiologist is tasked with the detection and ordinal grading of a single pathology type with three possible severity grades, which are enumerated as 0, 1, and 2. As illustrative examples, these simulated imaging exams could be considered to represent knee MRI in which radiologists assess the ACL to be either normal, moderately injured (e.g., partially torn), or severely injured (e.g., completely torn), or lumbar spine MRI in which central canal stenosis at a specific functional spinal unit is assessed to be not present or mild, moderate, or severe.

The predefined interpretive error rates of the radiologists were determined by two sets of simulation parameters: (1) the probability that pathology severity grades 0, 1, and 2 will be diagnosed correctly or if the pathology will be misdiagnosed as one of the other grades, respectively, and (2) the prevalence of imaging exams in which patients suffered from pathology grades 0, 1, and 2, respectively. The prevalence of exams with true pathology of grades 0, 1, and 2 was simulated to be 50%, 30%, and 20%, respectively. The “true” overall interpretive error rates defined for each of the 100 evaluated reading radiologists were 17% (i.e., in 83% of exams, the radiologist will grade the pathology correctly). Further, the evaluated radiologists’ rate of “two-degree errors,” which are defined to be errors where a grade 0 pathology is diagnosed as grade 2 or vice versa, is 3% (i.e., 97% of exams, the evaluated radiologist will grade the pathology in an exam correctly or be just one degree off). The prevalence and error rate values used in the simulation were derived from a real-world data set containing 33,989 studies interpreted by subspecialist radiologists. Naturally, prevalence and error rate values vary across pathologies, but the values in this study are representative.

The panel of three peer-reviewing radiologists in the simulated QI program were also assigned predefined probabilities to make interpretive errors in the same manner as the evaluated radiologist; however, three different profiles were defined for the reviewing radiologists with respect to the probabilities that they would correctly detect and grade the pathology of interest in the secondary peer reviews. Each peer-reviewing radiologist was assigned a profile with lower, equal, or higher probabilities of errors, Profiles 1 through 3, respectively, compared to what was defined for the evaluated radiologists. Reviewing radiologists assigned Profile 1 had an overall interpretive error rate of 13% and two-degree error rate of 2%; reviewing radiologists assigned Profile 3 had an overall interpretive error rate of 22% and two-degree error rate of 3%.

The complete details on the simulation’s implementation and the probabilities assigned to each type of interpretive error for evaluated and peer-reviewing radiologists are included in the Supplementary information 1.

### Interpretive Error Rate Measurement Using the BIRAR Method

The first step when using the peer review–based BIRAR method to measure interpretive error rates is the estimation of the error detection probability matrix (EDPM). The EDPM characterizes the conditional probabilities that an interpretive error of a specific type is present in a peer-reviewed exam, given that a specific discrepancy is flagged by a reviewing radiologist who performed a peer-review assessment. In this study, interpretive errors and discrepancies were categorized as either: no error, 1-degree undercall, 1-degree overcall, 2-degree undercall, or 2-degree overcall. Full details on the mathematical formulation and methodology used to calculate the EDPM are included in the Supplementary information 1 section, but the central aspect of the methodology is the use of Bayes’ theorem to calculate the probability of the patient’s true pathology grade, given: (1) the pathology grade reported by the initial reading radiologist, (2) the pathology grade assessed by the peer-reviewing radiologist, and (3) information about the rates that radiologists (both reading and peer reviewing) agree about various pathology grades. In this simulation study, the conditional probabilities contained in the EDPM are estimated using the results of 300 randomly selected exams, each peer-reviewed three times by radiologists randomly drawn from the pool of reviewing radiologists.

Once the EDPM is estimated, all QI program-sampled exams are subjected to a single peer review, but instead of using the results of the peer reviews to directly calculate interpretive error rates of the evaluated radiologist (i.e., treating each discrepancy as evidence of an interpretive error) the EDPM is used to transform the results of each peer review into a probability distribution over the five possible interpretive error statuses (i.e., no error, 1-degree undercall, 1-degree overcall, 2-degree undercall, and 2-degree overcall). These probability distributions can be aggregated across all the peer-reviewed exams related to each evaluated radiologist such that the expected value can be calculated for a radiologist’s overall rate of interpretive errors or the rate that interpretive errors of specific types occur (e.g., 2-degree undercalls and 2-degree overcalls). Full details on the mathematical formulation and methodology used to calculate interpretive error rates using the results of peer reviews and the EDPM are included in the Supplementary information 1.

In addition to error rate estimation, EDPM can be used to calculate sample size estimates. EPDM can be used to quantify the uncertainty in the data introduced by the inter-reviewer variability and thus allows us to propagate that uncertainty correctly into the interpretative error rate estimates of the evaluated radiologist. Therefore, sample size estimates can be produced to consider the impact of the noise in data due to inter-reviewer variability. As an example, when the inter-reviewer variability increases, the sample size will then be increased in order to keep the uncertainty of the interpretative error rates constant.

### Sensitivity Analyses

Two main sensitivity analyses were included in this study to evaluate the impact that different assumptions about the peer-reviewing radiologists’ error rates and different configurations of the BIRAR methodology would have on the overall study results.

The first sensitivity analysis evaluated the impact of using a different number of peer-reviewed exams to estimate the EDPM. The simulation described above used the results of 300 randomly selected exams, each peer-reviewed three times, to estimate the EDPM. In this sensitivity analysis, the simulation was rerun using the following alternate choices for the number of triple-peer-reviewed exams used to estimate the EDPM: 30, 50, 100, 300, 900, 2700.

The second sensitivity analysis evaluated the impact of the peer-reviewing radiologists’ error rates on the overall study results. In the simulation described above, the panel of reviewing radiologists was modeled to include, in equal amounts, radiologists with error rates that were lower than, equal to, and greater than the evaluated radiologists. For this sensitivity analysis, the simulation was rerun with two alternate configurations. The first alternate configuration set all of the reviewing radiologists to have error rate Profile 1, which as described above, defines a lower error rate than the evaluated radiologists. The second configuration set all of the reviewing radiologists to have error rate Profile 3, which as described above, defines a higher error rate than the evaluated radiologists.

## Results

**Fig. 3:**
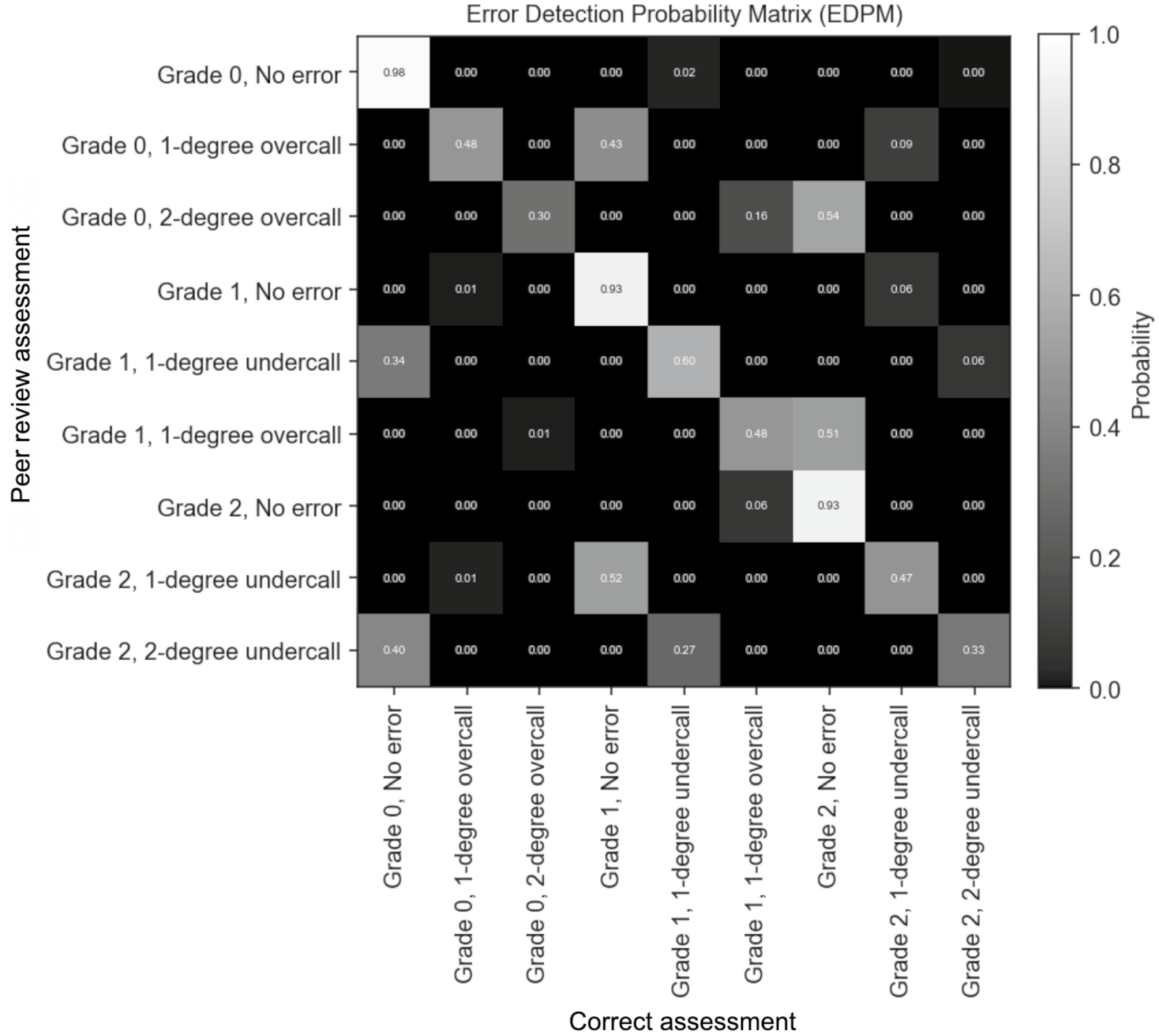
Error detection probability matrix (EDPM) that is calculated when using the data from 300 exams, each peer-reviewed three times. The pool of reviewing radiologists included, in equal amounts, radiologists who themselves had error rates lower than, equal to, and higher than the evaluated radiologists

The initial step using BIRAR for interpretive error rate measurement is the calculation of the EDPM (see Supplementary information 1). Figure 3 shows the resulting EDPM when using data from 300 exams, each peer-reviewed three times by radiologists who themselves had error rates that were either lower than, equal to, or higher than the evaluated radiologists (one-third of reviewing radiologists had each of the respective error rate profiles). The rows of the matrix indicate the peer-reviewing radiologist’s assessment, and the columns indicate the correct assessment for the exam; for example, the values in the fifth row indicate that if a reviewing radiologist records in a peer review that the pathology in an exam is Grade 1 and the initial reading radiologist committed a one-degree under call error (i.e., the evaluated radiologist reported Grade 0), there is a 60% probability that the peer-reviewing radiologist is correct (shown in column five), a 34% probability that the peer-reviewing radiologist is mistaken and the evaluated radiologist is correct (shown in column one), and a 6% probability that they are both mistaken and the correct finding for the exam is Grade 2 pathology. The probabilities in each row sum to one. It is important to note that the BIRAR method calculates the probabilities in the EDPM through statistical analysis of peer review–derived inter-reviewer agreement data and does not require any explicit information about the peer-reviewed exams’ true diagnosis (i.e., no gold standard diagnosis or consensus opinion is required).

**Fig. 4:**
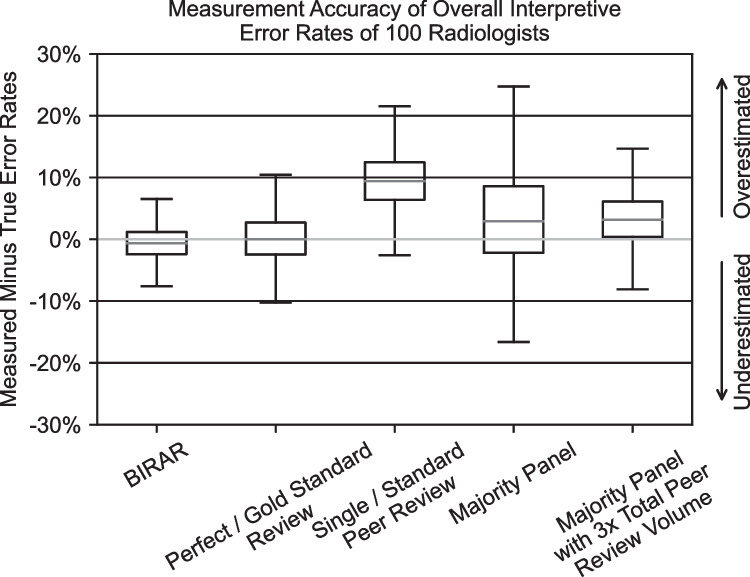
Boxplots of the difference between the measured and actual interpretive error rates of 100 radiologists using the five peer review–based error rate measurement methods simulated in this study. Standard boxplot notation is used with outliers not included in the graph. Boxplots are derived from 100 simulations

The results of the simulation of five methods for using peer reviews to measure the interpretive error rates of a population of 100 radiologists, with the peer-reviewing radiologists comprised of an equal number of radiologists with lower, equal, and higher error rates than the evaluated radiologists, are depicted in Fig. 4 and Table 1. As described in the “Materials and Methods” section, each of the evaluated reading radiologists had an actual overall interpretive error rate of 17%.

**Table 1:** Tabular summarization of results from the simulation of five methods for using peer review data to measure the overall interpretive error rates of 100 radiologists. Reported values are the median difference between the measured and actual interpretive error rates and the 95% credible interval (CI) around the median. Summary statistics are derived from 50 simulations

Interpretive error rate measurement method	Median difference (reported in percentage points) between measured and actual interpretive error rate and 95% CI
BIRAR	− 0.62 [− 5.64, 4.81]
Perfect/gold standard review	0.00 [− 6.88, 8.14]
Single/standard peer review	9.41 [0.67, 18.83]
Majority panel	2.91 [− 10.60, 20.12]
Majority panel with 3 × total peer review volume	3.15 [− 4.95, 11.98]

These results show that the BIRAR method is more accurate, as quantified by the median difference between the measured and actual interpretive error rates of the evaluated radiologists, compared to any of the other methods tested in this study except for the “Perfect / Gold Standard Reviewing” method, which is not a practical method to leverage in most QI programs due to the limited availability of gold standard diagnostic tests and given that “perfect peer reviewing radiologists” do not exist in the real world. The BIRAR measurements are 93.4% more accurate than the “Single / Standard Peer Review” method (median differences between the measured and actual interpretive error rates of − 0.62 versus + 9.41 percentage points, respectively) and 80.3% more accurate than the “Majority Panel with 3 × Total Peer Review Volume” method (median differences between the measured and actual interpretive error rates of − 0.62 versus + 3.15 percentage points, respectively).

Further, the accuracy of the BIRAR measurements displayed lower variability than all of the other methods including the “Perfect / Gold Standard Reviewing” method, as quantified by 95% CI around the median difference between the evaluated radiologists’ measured and actual interpretive error rates. The variability present in the “Perfect / Gold Standard Reviewing” interpretive error rate measurements is due to the fact that a finite sample of 90 peer-reviewed exams per evaluated radiologist is used to calculate interpretive error rates; the BIRAR method is able to reduce the measurement variability by 30.9% (i.e., 95% CI is 30.9% narrower) in the context of a QI program that aims to measure the interpretive error rates of 100 radiologists using 9000 total peer reviews. Similarly, the BIRAR method reduced the measurement variability by 42.5% and 66.0%, respectively, compared to what was observed using the “Single / Standard Peer Review” and “Majority Panel” methods.

**Fig. 5:**
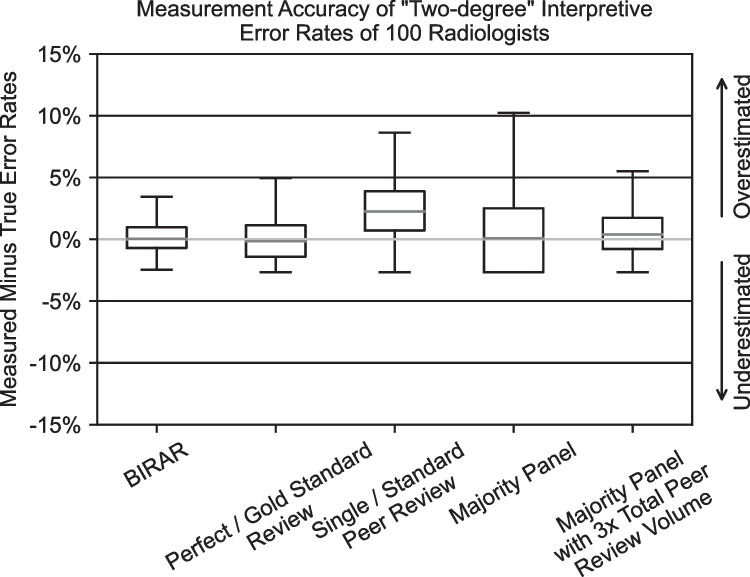
Boxplots of the difference between the measured and actual rates of “two-degree” interpretive errors for 100 radiologists using the five peer review–based error rate measurement methods simulated in this study. Standard boxplot notation is used with outliers not included in the graph. Boxplots are derived from 100 simulations

Figure 5 and Table 2 present a similar view of the study results, with the only difference being that the five-peer review–based methods were used to calculate the “two-degree” interpretive error rate of each evaluated radiologist. A “two-degree” interpretive error is when the reported finding indicates that a pathology is two degrees more or less severe than it actually is (i.e., a grade 0 pathology is reported as grade 2 or vice versa). As described in the “Materials and Methods” section, each of the evaluated radiologists was defined to have a two-degree interpretive error rate of 3%.

**Table 2:** Tabular summarization of results from the simulation of five methods for using peer review data to measure the “two-degree” interpretive error rates of 100 radiologists. Reported values are the median difference between the measured and actual interpretive error rates and the 95% credible interval (CI) around the median. Summary statistics are derived from 50 simulations

Interpretive error rate measurement method	Median difference (reported in percentage points) between measured and actual interpretive error rate and 95% CI
BIRAR	0.05 [− 1.79, 2.88]
Perfect/gold standard review	− 0.17 [− 2.67, 3.94]
Single/standard peer review	2.25 [− 1.61, 7.35]
Majority panel	0.08 [− 2.67, 9.08]
Majority panel with 3 × total peer review volume	0.39 [− 2.67, 4.74]

These results for the measurement of “two-degree” interpretive errors are consistent with the results presented above for the measurement of overall interpretive error rates. In this case, the simulation results demonstrated that the BIRAR method was more accurate, as quantified by the median difference between the evaluated radiologists’ measured and actual interpretive error rates, compared to all of the other methods tested, including the “Perfect / Gold Standard Reviewing” method. The BIRAR measurements are 97.8% more accurate than the “Single / Standard Peer Review” method (median differences between measured and actual interpretive error rates of + 0.05 versus + 2.25 percentage points, respectively) and 87.2% more accurate than the “Majority Panel with 3 × Total Peer Review Volume” method (median differences between measured and actual interpretive error rates of + 0.05 versus + 0.39 percentage points, respectively).

The BIRAR measurements of “two-degree” error rates also displayed lower variability than all of the other methods, as quantified by 95% CI around the median difference between the evaluated radiologists’ measured and actual interpretive error rates. The BIRAR method demonstrated 49.7% and 60.3% reductions in measurement variability compared to the “Single / Standard Peer Review” and “Majority Panel” methods, respectively.

**Fig. 6:**
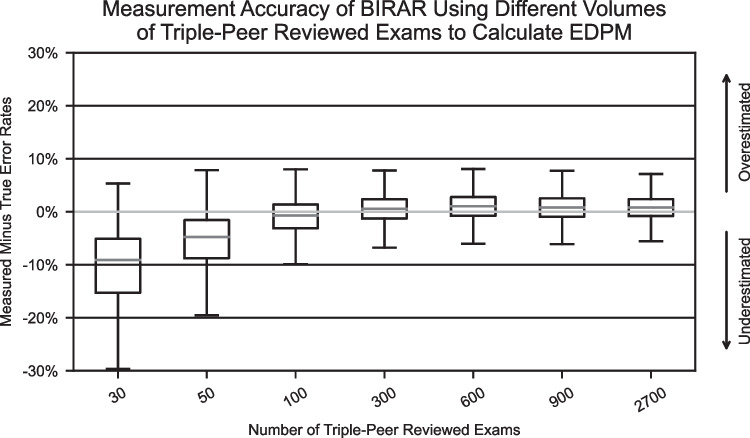
Boxplots of the difference between the BIRAR measured and actual interpretive error rates of 100 radiologists when using different volumes of triple-peer-reviewed exams to calculate the EDPM. Standard boxplot notation is used with outliers not included in the graph. Boxplots are derived from 100 simulations

The results of the sensitivity analysis to evaluate the impact of using a different number of peer-reviewed exams to estimate the EDPM are shown in Fig. 6. The simulation results show that when all other simulation configuration parameters remain constant, the measurement error of the BIRAR method, as quantified by the median difference between the evaluated radiologists’ measured and actual interpretive error rates, is approximately zero when using more than 100 triple-peer-reviewed exams. Modest additional reductions in the variability of the measurement accuracy are observed up to using 900 exams to calculate the EDPM.

**Fig. 7:**
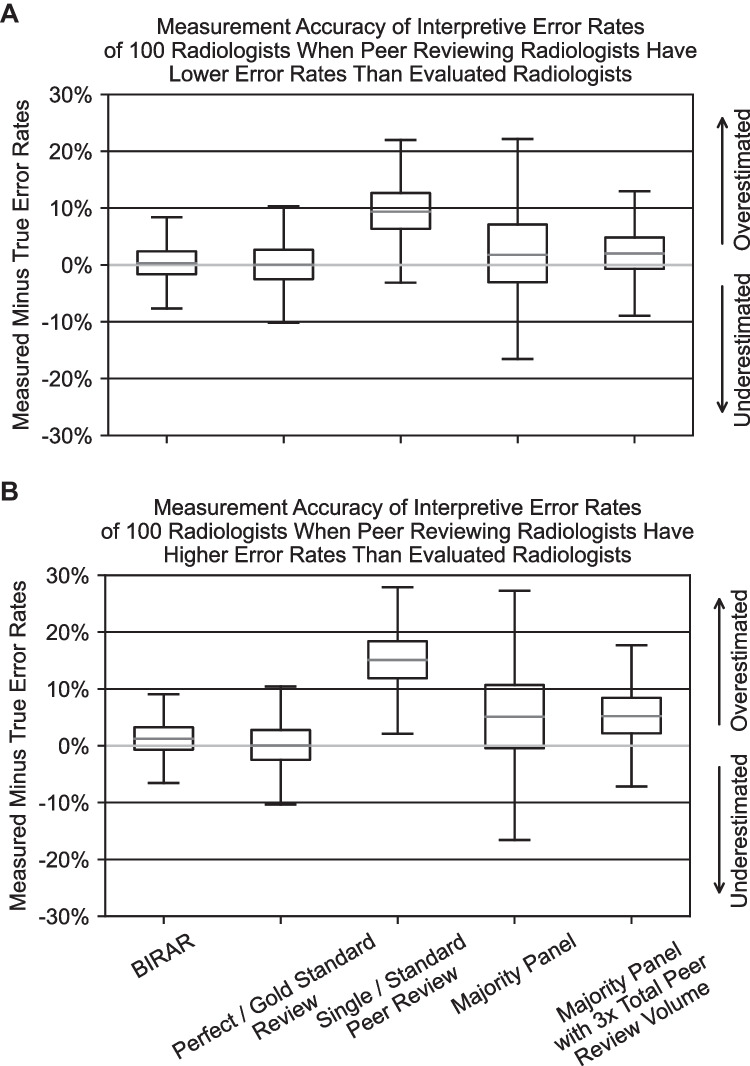
Boxplots of the difference between the measured and actual interpretive error rates of 100 radiologists using the five peer review–based error rate measurement methods simulated in this study. 7A shows the results simulated under conditions where the peer-reviewing radiologists have lower error rates than evaluated radiologists. 7B shows the results simulated under conditions where the peer-reviewing radiologists have higher error rates than evaluated radiologists. Standard boxplot notation is used with outliers not included in the graph. Boxplots are derived from 100 simulations

The results of the sensitivity analysis to evaluate the impact of the peer-reviewing radiologists’ error rates on the overall study results are shown below in Fig. 7 and Table 3. Figure 7A shows the results of the simulation of the five methods for using peer reviews to measure interpretive error rates of a population of 100 radiologists when the peer-reviewing radiologists all have a lower error rate than the evaluated radiologists (13% versus 17% overall error rate). Figure 7B shows the results of the same simulation with the reviewing radiologists’ error rates all defined to be higher than the evaluated radiologists (22% versus 17% overall error rate).

**Table 3:** Tabular summarization of results from the simulation of five peer review–based methods used to measure the overall interpretive error rates of 100 radiologists, simulated under conditions where the peer-reviewing radiologists have lower (middle column) and higher (rightmost column) error rates than evaluated radiologists. Reported values are the median difference between the measured and actual interpretive error rates and the 95% credible interval (CI) around the median

Interpretive error rate measurement method	Median difference (reported in percentage points) between measured and actual interpretive error rate and 95% CI	
	Reviewing radiologists’ error rates lower than evaluated radiologists	Reviewing radiologists’ error rates higher than evaluated radiologists
BIRAR	0.29 [− 5.39, 6.67]	1.22 [− 3.75, 7.96]
Perfect/gold standard review	0.06 [− 7.24, 7.92]	0.06 [− 7.24, 8.02]
Single/standard peer review	9.38 [0.84, 18.66]	15.09 [5.98, 24.71]
Majority panel	1.77 [− 11.24, 18.19]	5.09 [− 9.87, 22.77]
Majority panel with 3 × total peer review volume	2.02 [− 5.53, 10.49]	5.21 [− 3.33, 14.53]

The results of both sensitivity analyses show that the BIRAR method is more accurate even under conditions when peer-reviewing radiologists all have either lower or higher error rates than the evaluated radiologist, as quantified by the median difference between the measured and actual interpretive error rates of the evaluated radiologist, compared to all of the other methods tested in this study except for the “Perfect / Gold Standard Reviewing” method. When the peer-reviewing radiologists have lower error rates than the evaluated radiologists, BIRAR measurements are 96.9% more accurate than the “Single / Standard Peer Review” method, and 85.6% more accurate than the “Majority Panel with 3 × Total Peer Review Volume.” When the peer-reviewing radiologists have higher error rates than the evaluated radiologists, BIRAR measurements are 91.9% more accurate than the “Single / Standard Peer Review” method, and 76.6% more accurate than the “Majority Panel with 3 × Total Peer Review Volume.”

Similarly, the accuracy of the BIRAR measurements again displayed lower variability than all of the other methods, as quantified by 95% CI around the median difference between the evaluated radiologists’ measured and actual interpretive error rates. When the peer-reviewing radiologists have lower error rates than the evaluated radiologists, BIRAR reduced the measurement variability by 20.4%, 32.3%, and 59.0%, respectively, compared to the “Perfect / Gold Standard Reviewing,” “Single / Standard Peer Review,” and “Majority Panel” methods. When the peer-reviewing radiologists have higher error rates than the evaluated radiologists, BIRAR reduced the measurement variability by 23.3%, 37.5%, and 64.1%, respectively, compared to the “Perfect / Gold Standard Reviewing,” “Single / Standard Peer Review,” and “Majority Panel” methods.

## Discussion

QI programs need practical strategies that provide meaningful and robust measures of quality in order to better support peer learning and quality improvement efforts. BIRAR is novel because it uses a small number of exams that are subjected to multiple peer reviews to incorporate inter-reviewer variation into the metric, which yields better accuracy, lower variability, and practicality. In the sections below, the ways in which this strategy improves accuracy and robustness are discussed first, and then the method is placed in the context of current common strategies for peer review to show practicality. Finally, limitations are discussed.

### BIRAR Produces Accurate and Robust Measurements

In the simulation study, the “Single / Standard Peer Review” and “Majority Panel Peer Review” methods demonstrated a consistent tendency to overestimate interpretive error rates: the median difference between the estimated and actual interpretive error rates for the “Single / Standard Peer Review” method was + 9.4% (55.4% overestimation); for the “Majority Panel Peer Review” method, it was + 2.9% (17.1% overestimation); and for the BIRAR method, − 0.62% (3.6% underestimation). The reason for the overestimation is that the errors made by the peer-reviewing radiologists are accounted for by the evaluated radiologists. Even though the overestimation bias is smaller with the “Majority Panel Peer Review” method, it is not negligible with three reviewers as the consensus opinion is still sometimes wrong. In addition, the “Single / Standard Peer Review” method’s 95% CI range was completely positive, indicating that error rates were overestimated for 100% of evaluated radiologists. The “Majority Panel Peer Review” method’s 95% CI was 66% positive, while BIRAR appeared more balanced, overestimating 54% and underestimating 46%.

As expected, the performance of all of the interpretive error rate measurement methods was degraded when simulating peer-reviewing radiologists with higher error rates than the evaluated radiologists, though the BIRAR proved to be more robust: “Single / Standard Peer Review” + 15.09% (95% CI 5.98–24.71); “Majority Panel Peer Review” + 5.09% (95% CI − 9.87–22.77); BIRAR + 1.22% (95% CI − 3.75–7.96). Thus, BIRAR can be used in a variety of practice settings, including those where “expert” reviewers are not available. This is a direct consequence of incorporating information from inter-reviewer agreement rates into the metric.

**Fig. 8:**
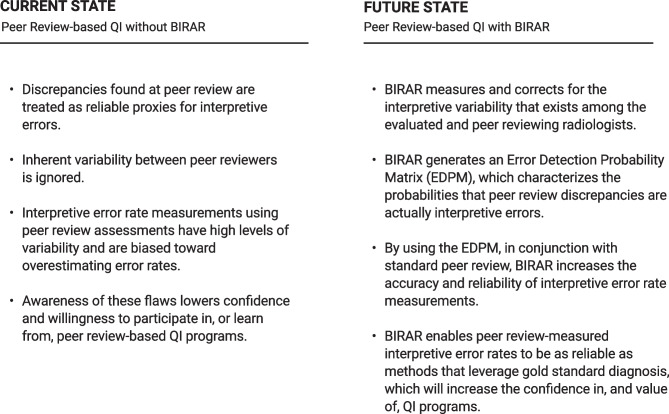
Comparison of the current state of peer review in QI programs to a “future state” in which BIRAR is used to improve the accuracy and reliability of peer review

In general, BIRAR offers important enhancements to the most common current state implementations of peer review (Fig. 8). Specific enhancements vary by the type of program to which it is compared:

#### Gold Standard Follow-Up Tests

BIRAR is a practical alternative to using a gold standard because it may be employed to assess any exam currently undergoing peer review. In practice, a gold standard strategy is not viable because (1) only a limited subset of radiology exam types and patient studies occur prior to gold standard diagnostic tests, (2) generally the gold standard follow-up test will only be performed after an initial positive radiology finding, which limits the ability for this approach to detect false negatives, and (3) the gold standard tests can also suffer from errors, which reduces the error measurement reliability.

#### Single Review

Single reviewer discrepancy rates have been shown to be unreliable measures of actual rates of interpretive error and agreement among peer reviewers is poor [5, 13, 15, 16]. BIRAR addresses both shortcomings, as its interpretive error rate measurements are more accurate, and this enhanced accuracy is directly because the method specifically incorporates inter-reviewer variability.

#### Panel Review

Panel review has been proposed as a solution to reducing the impact of variation between reviewers [5, 17]. For example, some mammography quality assurance and improvement programs assign exams initially classified as negative to three independent secondary peer reviews; if two of three radiologists concur on the presence and location of cancer, this is considered evidence of a false negative interpretive error [18]. Approaches like these may yield more reliable measures of interpretive error rates compared to a single review, but the requirement for multiple secondary assessments or dedicated consensus discussions creates significant demands on radiologists’ time and resources. BIRAR incorporates features of panel review, with only a relatively small subset of exams assessed by more than one reviewer and removing the requirement for peer reviewers to reach consensus. Again, in this simulation study, BIRAR’s performance exceeded that of the “Majority Panel with 3 × Total Peer Review Volume” method, even though it required less total peer reviews to measure the interpretive error rates of the evaluated radiologists.

### Uncertainty in Radiology: When Is an Error Not an Error?

Practicing radiologists (and indeed all physicians) daily face inherent variations in disease presentation, natural history, and imaging appearance that often makes diagnosis uncertain. In fact, experienced thoracic subspecialists may disagree on pulmonary nodule characterization [19], and radiologists may come to different classifications when applying well-defined standards such as TIRADS [20] or PIRADS [21] or obtain different results when performing seemingly straightforward size measurements on PACS [22, 23]. Disagreement between radiologists does not necessarily indicate an interpretive error has occurred, but instead, it has been proposed that variation in interpretation may at times be more accurately described as “differing opinions” in the face of an unclear diagnosis [2]. By not incorporating measurements of peer reviewer variation, most QI programs do not formally acknowledge the unavoidable uncertainty that radiologists face, and this may be one cause for radiologists’ lack of confidence in quality metrics. In contrast, instead of calculating a simple interpretive error rate, BIRAR uses a small number of triple-peer-reviewed studies to also calculate a confidence interval for the error rate. If a particular diagnosis tends to be difficult with much disagreement (variation) among radiologists (peer reviewing and reading), this will contribute to a wide confidence interval and low probabilities in the EDPM associated with the likelihood of an error in the initial interpretation given a peer review discrepancy. Therefore, in this case, a difference in interpretation between peer-reviewing and initial-reading radiologists for difficult diagnoses would be recorded, but the BIRAR method accounts for the uncertainty and will not classify such discrepancies as errors.

### BIRAR in Practice

Realizing the benefits of BIRAR requires standardized data collection, data tabulation, statistical analysis, and multiple peer reviews. Ideally, BIRAR should be implemented in practice using structured templated reports. Utilization of structured reports can facilitate the identification of inter-reviewer variability for specific pathologies from a set of studies reviewed by multiple reviewers. BIRAR can be most useful for pathologies that exhibit relatively high inter-reader variability, and applying BIRAR to these pathologies may result in increased confidence in the peer-review process. Data pipelines have to be implemented to ingest and analyze peer-review data using BIRAR on an ongoing basis. Some (but not all) of these requirements are already inherent in existing quality programs, but BIRAR will likely require additional time, resources, and statistical expertise. For instance, translating the output of BIRAR into actionable and useful feedback will require additional work and is outside of the scope of this study. It should be noted that BIRAR can be deployed with alternative configurations that may better suit the needs of a particular institution. EDPM used in BIRAR has to be estimated for each pathology type separately since inter-reviewer variability is pathology-type specific. For example, BIRAR was presented in this study as a two-step process, with a smaller number of triple-peer-reviewed studies followed by a larger number of single peer-reviewed studies, but instead, sites may intersperse or periodically collect the data from multiple-peer-reviewed exams. Importantly, if the pool of experts or their performance changes significantly over time, then a new set of triple-reviewed study data should be collected, and the inter-reviewer variability (i.e., EPDM) should be recalculated. Notably, if peer-reviewing radiologists are selected for each study from a large pool of radiologists with varying performance levels, then it can be difficult to get a representative sample for estimating inter-reviewer variability accurately. That is, BIRAR would be easiest to deploy in settings where the panel of experts is relatively small, and it does not change over time. Finally, BIRAR can be deployed in a targeted manner only assessing a particular study type or specific pathology, and it can also be used in a research context or to improve the selection of studies for peer learning.

## Limitations

While simulation studies can fail to capture all aspects of how a methodology may work in the “real world,” the authors believe that evaluating the performance of this novel approach using simulated peer review data was the best choice because it allowed explicit and precise comparisons to be made between the interpretive error rates calculated using various peer review–based measurement methods and the actual “true” interpretive error rate of hypothetical radiologists under evaluation, which were predetermined within the simulation. A study that instead used real patient exams and peer review data would suffer from several complicating issues, including uncertainty related to the gold standard(s) used, unknown evaluated and peer-reviewing radiologist error rates, and/or the potential lack of generalizability of the study’s results to other clinical contexts where interpretive error rates and their ability to be reliably flagged through secondary assessments may vary. BIRAR has limited value in areas of imaging where inter-reviewer variability is low. The authors made efforts to choose realistic values to calibrate the simulation and to also investigate the sensitivity of the results to important calibration parameters, like the relative error rates of the peer-reviewing and evaluated radiologists. Real-world constraints can limit BIRAR performance when introduced into clinical practice, but the current study provides value because simulations are often precursors to real-world implementation. Finally, the method described in this study covers the analysis of the data collected in quality programs while taking into account inter-reviewer variability, but it does not address how quality programs should be implemented in practice.

## Conclusions

BIRAR can increase the value of peer review within QI programs by enabling more accurate and less variable peer review–based quality measures. These quality measures can be useful for identifying outlying providers who generate reports that diverge from the accepted norm of the group’s collective interpretation standards. The BIRAR method’s statistical approach allows for a more standardized assessment of quality even when there is variability in the sensitivity of the radiologists performing peer reviews. BIRAR enables QI programs to assess interpretive accuracy without relying on gold standard comparison tests by implicitly deriving a standard from the levels of consensus within the group across various types of interpretive findings; however, multiple peer review assessments or consensus discussions are not required for every study evaluated by the QI program. These features allow this approach to be scaled across a large population of radiologists and practically implemented in a group’s QI program.

### Supplementary Information

Below is the link to the electronic supplementary material.

Supplementary file1 (PDF 103 KB)

## Data Availability

A Stan implementation of the generative model is available in Supplementary Information.

## Abbreviations

QI: Quality improvement
BIRAR: Bayesian inter-reviewer agreement rate
EDPM: Error detection probability matrix
