# Supplementary material for: Improving the Reliability of Peer Review Without a Gold Standard
Source: J Imaging Inform Med. 2024 Feb 5;37(2):489–503. doi: 10.1007/s10278-024-00971-9 (PMC11031531; doi:10.1007/s10278-024-00971-9)
Supplement: Supplementary file 1 — Supplementary file1 (PDF 103 KB) [file 10278_2024_971_MOESM1_ESM.pdf]

Improving the Reliability of Peer Review Without a Gold Standard

October 25, 2023

Notations

Let us assume there are N_{grades} different severity grade levels. Let us define the set of G severity grade levels as $\mathcal{G} = \{0, 1, 2, N_{\text{grades}} - 1\}$.

Let us consider five discrepancy values

$$y \in \{\text{Agree}, \text{1-degree undercall}, \text{1-degree overcall}, \text{2-degree undercall}, \text{2-degree overcall}\}. \tag{1}$$

The discrepancy value is defined in terms of $o \in \mathcal{G}$ (grade given by QA'd Radiologist) and $r \in \mathcal{G}$ (grade given by QA'ing Radiologist) as follows

$$f(o,r) = \begin{cases} \text{Agree} & \text{if } o = r \\ \text{1-degree undercall} & \text{if } o + 1 = r \\ \text{1-degree overcall} & \text{if } o - 1 = r \\ \text{2-degree undercall} & o + 1 < r \\ \text{2-degree overcall} & o - 1 > r. \end{cases} \tag{2}$$

That is, the discrepancy value as defined in Equation (2) captures the discrepancy of o with respect to r.

Let there be N_{studies} and $N_{\text{reviewers}}$ studies and reviewers, respectively.

Generative model

Definition

Next, we formulate a hierarchical model for o as follows

$$\begin{aligned} p^{\text{QA'd}} | \alpha^{\text{QA'd}} &\sim \text{Dirichlet}(\alpha^{\text{QA'd}}), \\ o | p^{\text{QA'd}} &\sim \text{Categorical}(p^{\text{QA'd}}), \end{aligned} \tag{3}$$

where $\alpha^{\text{QA'd}}$ is a user-defined parameter.

Similarly, we formulate a hierarchical model for r as follows

$$\begin{aligned} p^{\text{QA'ing}} | \alpha^{\text{QA'ing}} &\sim \text{Dirichlet}(\alpha^{\text{QA'ing}}), \\ r | p^{\text{QA'ing}} &\sim \text{Categorical}(p^{\text{QA'ing}}), \end{aligned} \tag{4}$$

where $\alpha^{\text{QA'ing}}$ is a user-defined parameter.

We consider N_{grades} patient profiles. Therefore, we formulate a hierarchical model for patient profile, $s \in 0, 1, \ldots, N_{\text{grades}} - 1$, as follows

$$s | \mathbf{p}^S \sim \text{Categorical}(\mathbf{p}^S) \tag{5}$$

where $\mathbf{p}^S$ is a user-defined parameter.

Next, by combining the previous models, we can formulate a hierarchical model for y as follows

$$\begin{aligned} s_i | \mathbf{p}^s &\sim \text{Categorical}(\mathbf{p}^s),\ i = 1, 2, \ldots, N_{\text{studies}}, \\ \mathbf{p}_i^{\text{QA'd}} | \alpha_{s_i}^{\text{QA'd}} &\sim \text{Dirichlet}(\alpha_{s_i}^{\text{QA'd}}),\ i = 1, 2, \ldots, N_{\text{studies}}, \\ o_i | \mathbf{p}_i^{\text{QA'd}} &\sim \text{Categorical}(\mathbf{p}_i^{\text{QA'd}}),\ i = 1, 2, \ldots, N_{\text{studies}}, \\ \mathbf{m}_i &= \text{Permutate}\left((1, 2, \ldots, N_{\text{QA'ing radiologists}})\right), \\ \mathbf{p}_{i,j}^{\text{QA'ing}} | \alpha_{s_i}^{\text{QA'ing}, m_{i,j}} &\sim \text{Dirichlet}(\alpha_{s_i}^{\text{QA'ing}, m_{i,j}}),\ i = 1, 2, \ldots, N_{\text{studies}}, j = 1, 2, \ldots, N_{\text{reviewers}}, \\ r_{i,j} | \mathbf{p}_{i,j}^{\text{QA'ing}} &\sim \text{Categorical}(\mathbf{p}_{i,j}^{\text{QA'ing}}),\ i = 1, 2, \ldots, N_{\text{studies}}, j = 1, 2, \ldots, N_{\text{reviewers}}, \\ y_{i,j} &= f(o_i, r_{i,j}),\ i = 1, 2, \ldots, N_{\text{studies}}, j = 1, 2, \ldots, N_{\text{reviewers}}. \end{aligned} \tag{6}$$

Note that $\mathbf{m}$ is used to permutate the order of QA'ing Radiologists for each study separately. This is needed so that in the case of N_{reviews} less than the number of QA'ing Radiologists, the studies are not always reviewed by the same subset of QA'ing Radiologists.

Graphical model

The model defined in Equation (6) is visualized in Figure 1.

Data simulation

The model defined in Equation (6) is used to simulate QA review data. The rates of diagnostic errors defined for the QA'd Radiologist and the relative rates that QA'd Exams will have true pathology grades 0, 1, and 2, were roughly informed by empirical data observed by the authors in active QA programs, but are effectively arbitrary choices that enable the relative performance of the five diagnostic error rate measurement methodologies to be compared to each other. In the following sections, we describe in detail how we calibrated the generative model for simulating data.

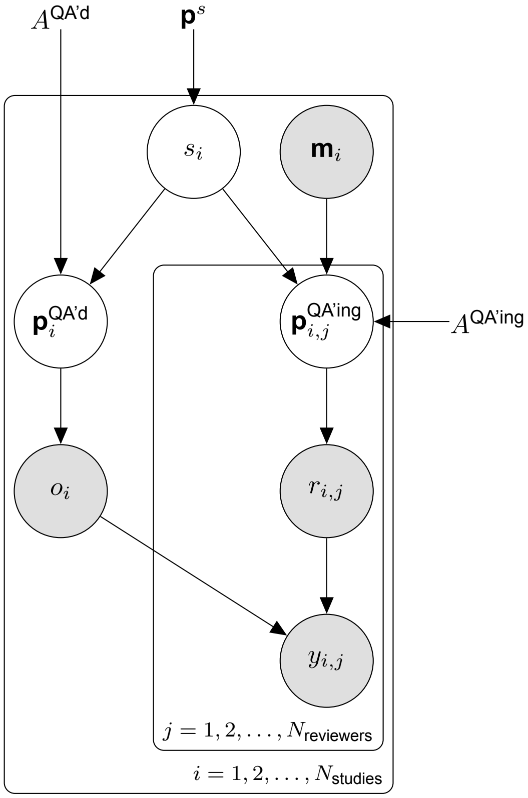

Figure 1: The generative model defined Equation (6) is visualized using the plate notation. The grey and white circles denote observed and latent variables, respectively. The solid black circles denote user-definable parameters. The plates denote repetitions. The directed edges indicate dependencies between variables.

Calibration of QA'd exams

The probabilities that QA'd Exams will have true pathology grades equal to 0, 1, and 2, respectively, were calibrated using the three probabilities defined in the vector $\mathbf{p}^s$ as follows

$$\mathbf{p}^s = \begin{pmatrix} 0.5 & 0.3 & 0.2 \end{pmatrix}^{\mathsf{T}}. \tag{7}$$

The vector $\mathbf{p}^s$ can be interpreted as "50% of the QA'd Exams have pathology of grade 0, 30% have pathology of grade 1, and 20% have pathology of grade 2."

Calibration of interpreting radiologists

The predefined probabilities that patient exams with true pathologies of specific grades would be interpreted as the correct grade or incorrectly as one of the other grades, are modeled as Dirichlet distributions, which are calibrated using the following pattern of correct and incorrect diagnoses defined in the matrix $A^{\text{QA'd}}$ as follows

$$A^{\text{QA'd}} = \begin{pmatrix} \alpha_0^{\text{QA'd}^{\mathsf{T}}} \\ \alpha_1^{\text{QA'd}^{\mathsf{T}}} \\ \alpha_2^{\text{QA'd}^{\mathsf{T}}} \end{pmatrix} = \begin{matrix} \\ s=0 \\ s=1 \\ s=2 \end{matrix} \begin{matrix} o=0 & o=1 & o=2 \\ \begin{pmatrix} 80 & 4 & 2 \\ 3 & 15 & 3 \\ 2 & 4 & 20 \end{pmatrix} \end{matrix}. \tag{8}$$

Where the first row in $A^{\text{QA'd}}$ can be interpreted as, "out of 86 exams in which the true grading of the patient's pathology is '0', the QA'd Radiologist will correctly grade the exam as '0' 80 times, incorrectly grade the exam as grade '1' 4 times, and incorrectly grade the exam as '2' 2 times." The second row in $A^{\text{QA'd}}$ can be interpreted as, "out of 21 exams in which the true grading of the patient's pathology is '1', the QA'd Radiologist will correctly grade the exam as '1' 15 times, incorrectly grade the exam as grade '0' 3 times, and incorrectly grade the exam as '2' 3 times." The third row in $A^{\text{QA'd}}$ can be interpreted as, "out of 26 exams in which the true grading of the patient's pathology is '2', the QA'd Radiologist will correctly grade the exam as '2' 20 times, incorrectly grade the exam as grade '0' 2 times, and incorrectly grade the exam as '1' 4 times."

The overall interpretive error rate assigned to each of the QA'd radiologists is predefined to be 17% (i.e. in 83% of exams, the QA'd radiologist will grade the pathology correctly). Further, the QA'd radiologists' rate of "two-degree errors", which are defined to be errors where a grade 0 pathology is diagnosed as grade 2 or vice versa, is 3% (i.e. 97% of exams, the QA'd radiologist will grade the pathology in an exam correctly or be just one degree off).

Calibration of reviewing radiologists

The panel of reviewing radiologists participating in the simulated QA program were assigned predefined probabilities to make interpretive errors in the same

manner as the QA'd radiologists, however they were defined to have three different profiles with respect to the probabilities that they would correctly detect and grade the pathology of interest in the secondary QA reviews. These probabilities were modeled in the same manner as the QA'd Radiologists, described above, and were calibrated using the patterns of correct and incorrect diagnoses defined in the following three matrices $A^{\text{QA'ing},1}$, $A^{\text{QA'ing},2}$, and $A^{\text{QA'ing},3}$ as follows

$$A^{\text{QA'ing},1} = \begin{pmatrix} \alpha_0^{\text{QA'ing},1^{\mathsf{T}}} \\ \alpha_1^{\text{QA'ing},1^{\mathsf{T}}} \\ \alpha_2^{\text{QA'ing},1^{\mathsf{T}}} \end{pmatrix} = \begin{matrix} \\ s=0 \\ s=1 \\ s=2 \end{matrix} \begin{matrix} \begin{matrix} r=0 & r=1 & r=2 \end{matrix} \\ \begin{pmatrix} 100 & 4 & 2 \\ 3 & 20 & 3 \\ 2 & 4 & 25 \end{pmatrix} \end{matrix}, \tag{9}$$

$$A^{\text{QA'ing},2} = \begin{pmatrix} \alpha_0^{\text{QA'ing},2^{\mathsf{T}}} \\ \alpha_1^{\text{QA'ing},2^{\mathsf{T}}} \\ \alpha_2^{\text{QA'ing},2^{\mathsf{T}}} \end{pmatrix} = \begin{matrix} \\ s=0 \\ s=1 \\ s=2 \end{matrix} \begin{matrix} \begin{matrix} r=0 & r=1 & r=2 \end{matrix} \\ \begin{pmatrix} 80 & 4 & 2 \\ 3 & 15 & 3 \\ 2 & 4 & 20 \end{pmatrix} \end{matrix}, \tag{10}$$

and

$$A^{\text{QA'ing},3} = \begin{pmatrix} \alpha_0^{\text{QA'ing},3^{\mathsf{T}}} \\ \alpha_1^{\text{QA'ing},3^{\mathsf{T}}} \\ \alpha_2^{\text{QA'ing},3^{\mathsf{T}}} \end{pmatrix} = \begin{matrix} \\ s=0 \\ s=1 \\ s=2 \end{matrix} \begin{matrix} \begin{matrix} r=0 & r=1 & r=2 \end{matrix} \\ \begin{pmatrix} 60 & 4 & 2 \\ 3 & 10 & 3 \\ 2 & 4 & 15 \end{pmatrix} \end{matrix}. \tag{11}$$

Inspection of these three calibration matrices reveals that Reviewing Radiologist Profiles 1 (defined by $A^{\text{QA'ing},1}$), 2 (defined by $A^{\text{QA'ing},2}$), and 3 (defined by $A^{\text{QA'ing},3}$), are defined to have lower, equal, and higher probabilities of errors, respectively, compared to what was defined for the QA'd Radiologists above. Given $\mathbf{p}^s$ as defined above, the diagnostic error rates of the Reviewing Radiologist with assigned Profile 1 had an overall interpretive error rate of 13% and reviewing radiologists assigned Profile 3 had an overall interpretive error rate of 22%, respectively.

Estimation of Reviewer Error Detection Probability Matrix (REDPM)

Generative model

We use a simplified version of the model defined in Equation (6) as follows

$$
\begin{aligned}
\mathbf{p}^S &\sim \text{Dirichlet}(\mathbf{1}),\\
\mathbf{p}_k^{\text{QA'd}} &\sim \text{Dirichlet}(\mathbf{1}),\ k = 0, 1, \ldots, N_{\text{grades}},\\
\mathbf{p}_k^{\text{QA'ing}} &\sim \text{Dirichlet}(\mathbf{1}),\ k = 0, 1, \ldots, N_{\text{grades}},\\
s_i|\mathbf{p}^S &\sim \text{Categorical}(\mathbf{p}^S),\ i = 1, 2, \ldots, N_{\text{studies}},\\
o_i|\mathbf{p}_{s_i}^{\text{QA'd}} &\sim \text{Categorical}(\mathbf{p}_{s_i}^{\text{QA'd}}),\ i = 1, 2, \ldots, N_{\text{studies}},\\
r_{i,j}|\mathbf{p}_{s_i}^{\text{QA'ing}} &\sim \text{Categorical}(\mathbf{p}_{s_i}^{\text{QA'ing}}),\ i = 1, 2, \ldots, N_{\text{studies}}, j = 1, 2, \ldots, N_{\text{reviewers}},\\
y_{i,j} &= f(o_i, r_{i,j}),\ i = 1, 2, \ldots, N_{\text{studies}}, j = 1, 2, \ldots, N_{\text{reviewers}}.
\end{aligned}
\tag{12}
$$

Note that in the model defined in Equation (12) we do not have the hierarchical priors for $p_k^{\text{QA'd}}$ and $p_k^{\text{QA'ing}}$ as in Equation (6) , and additionally, $\mathbf{p}_k^{\text{QA'ing}}$ is shared across QA'ing Radiologists. That is, we will estimate a single REPDM; however, we could estimate REDPM for each QA'ing Radiologist separately.

The posterior probability density function of the model defined Equation (12) is proportional to the joint probability density function

$$
\begin{aligned}
& p(\theta|\mathcal{D}) \propto p(\mathcal{D}, \theta) = p(\mathcal{D}|\theta)p(\theta)\\
&= \prod_{i=1}^{N_{\text{studies}}} \left[\sum_{s \in \mathcal{G}} \left[\text{Categorical}\left(s|\mathbf{p}^S\right) \text{Categorical}\left(o_i|\mathbf{p}_s^{\text{QA'd}}\right) \prod_{j=1}^{N_{\text{reviews}}} \text{Categorical}\left(r_{i,j}|\mathbf{p}_s^{\text{QA'ing}}\right) \right] \right]\\
&\quad \left[\prod_{s \in \mathcal{G}} \text{Dirichlet}\left(\mathbf{p}_s^{\text{QA'd}}|\mathbf{1}\right) \text{Dirichlet}\left(\mathbf{p}_s^{\text{QA'ing}}|\mathbf{1}\right) \right] \text{Dirichlet}\left(\mathbf{p}^S|\mathbf{1}\right),
\end{aligned}
\tag{13}
$$

where $\mathcal{D} = \{(o_1, (r_{1,1}, \ldots, r_{1,N_{\text{reviews}}})), \ldots, (o_{N_{\text{studies}}}, (r_{N_{\text{studies}},1}, \ldots, r_{N_{\text{studies}},N_{\text{reviews}}}))\}$. Note that the discrete variables $s_i,\ i = 1, 2, \ldots, N_{\text{studies}}$ are marginalized out.

QA'ing Radiologists' error detection probability

We can state the probability of s given o and r under the model defined in Equation (12) using the Bayes' theorem as follows

$$\begin{aligned} p(s|o,r) &= \frac{p(o,r|s)p(s)}{p(o,r)} \\ &= \frac{p(o,r|s)p(s)}{\sum_{s'\in\mathcal{G}} p(o,r|s')p(s')} \\ &= \frac{p(o|s)p(r|s)p(s)}{\sum_{s'\in\mathcal{G}} p(o|s')p(r|s')p(s')} \\ &= \frac{p^{\text{QA'd}}_{s,o} p^{\text{QA'ing}}_{s,r} p^{S}_{s}}{\sum_{s'\in\mathcal{G}} p^{\text{QA'd}}_{s',o} p^{\text{QA'ing}}_{s',r} p^{S}_{s'}} \end{aligned} \tag{14}$$

These probabilities quantify our belief in the grade levels, r, given by QA'ing Radiologists.

For instance, let

$$\begin{aligned} N_{\text{grades}} = 3,\ \mathbf{p}^{S} = \begin{pmatrix}0.5 & 0.3 & 0.2\end{pmatrix}^{\mathsf{T}}, \\ \mathbf{p}^{\text{QA'd}}_{0} = \begin{pmatrix}0.85 & 0.1 & 0.05\end{pmatrix}^{\mathsf{T}},\ \mathbf{p}^{\text{QA'd}}_{1} = \begin{pmatrix}0.2 & 0.6 & 0.2\end{pmatrix}^{\mathsf{T}},\ \mathbf{p}^{\text{QA'd}}_{2} = \begin{pmatrix}0.1 & 0.25 & 0.65\end{pmatrix}^{\mathsf{T}}, \\ \mathbf{p}^{\text{QA'ing}}_{0} = \begin{pmatrix}0.9 & 0.075 & 0.025\end{pmatrix}^{\mathsf{T}},\ \mathbf{p}^{\text{QA'ing}}_{1} = \begin{pmatrix}0.15 & 0.7 & 0.15\end{pmatrix}^{\mathsf{T}},\ \mathbf{p}^{\text{QA'ing}}_{2} = \begin{pmatrix}0.1 & 0.2 & 0.7\end{pmatrix}^{\mathsf{T}}, \end{aligned} \tag{15}$$

then

$$p(s|o,r) \approx \begin{array}{c} \begin{matrix} s=0 & s=1 & s=2 \end{matrix} \\ \begin{pmatrix} 0.972 & 0.023 & 0.005 \\ 0.409 & 0.539 & 0.051 \\ 0.316 & 0.268 & 0.416 \\ 0.584 & 0.351 & 0.065 \\ 0.027 & 0.902 & 0.072 \\ 0.020 & 0.427 & 0.553 \\ 0.506 & 0.601 & 0.292 \\ 0.027 & 0.601 & 0.372 \\ 0.006 & 0.089 & 0.904 \end{pmatrix} \end{array} \begin{array}{l} \\ o=0,r=0 \\ o=0,r=1 \\ o=0,r=2 \\ o=1,r=0 \\ o=1,r=1 \\ o=1,r=2 \\ o=2,r=0 \\ o=2,r=1 \\ o=2,r=2 \end{array} \tag{16}$$

Using a similar approach as in Equation (14), we can state the probabilities $p\left((s, f(o,s))\,|\,(r, f(o,r))\right)$. If the discrepancy function $f(x,\cdot)$ for some x is a many-to-one function, i.e. $\exists x \in \mathcal{G}\, \exists y,z \in \mathcal{G}\, (f(x,y) = f(x,z) \wedge y \neq z)$, then we might want to aggregate over those equivalence classes as our focus is on the pairs made of a severity grade and a discrepancy value. These probabilities quantify our belief in the grade levels and discrepancy values, $(r, f(o,r))$, given by QA'ing Radiologists.

Inference

We can implement the model defined in Equation (12) in Stan as shown in Listing 1.

Listing 1: A Stan implemention of the model defined in Equation (12)

```
data {
  int<lower=1> N_studies;
  int<lower=1> N_reviews;
  int<lower=1> N_grades;
  int<lower=1,upper=N_grades> r[N_studies,N_reviews];
  int<lower=1,upper=N_grades> o[N_studies,1];
}
transformed data {
  vector[N_grades] alpha_p_qad = rep_vector(1,N_grades);
  vector[N_grades] alpha_p_qaing = rep_vector(1,N_grades);
  vector[N_grades] alpha_p_s = rep_vector(1,N_grades);
}
parameters {
  simplex[N_grades] p_s;
  simplex[N_grades] p_qad[N_grades];
  simplex[N_grades] p_qaing[N_grades];
}
model {
  vector[N_grades] tmp;

  p_s ~ dirichlet(alpha_p_s);

  for (j in 1:N_grades) {
    p_qad[j] ~ dirichlet(alpha_p_qad);
    p_qaing[j] ~ dirichlet(alpha_p_qaing);
  }

  for (i in 1:N_studies) {
    for (j in 1:N_grades) {
      tmp[j] = log(p_s[j])+categorical_lpmf(o[i]|p_qad[j])
                          +categorical_lpmf(r[i]|p_qaing[j]);
    }
    target += log_sum_exp(tmp);
  }
}
```

Stan produces samples from the density $p(\theta|\mathcal{D})$, that is we obtain samples $\mathbf{p}^{S^{(i)}}, \mathbf{p}_k^{\text{QA'd}^{(i)}}, \mathbf{p}_k^{\text{QA'ing}^{(i)}}$, where $k = 1, 2, \ldots, N_{\text{grades}}$ and $i = 1, 2, \ldots, N_{\text{samples}}$. Using these posterior samples we can produce posterior samples of $p(s|o,r)^{(i)}$ and $p\left((s, f(o,s)) \,|\, (r, f(o,r))\right)^{(i)}$, where $i = 1, 2, \ldots, N_{\text{samples}}$. In practice, we

estimate $\mathbb{E}[p(s|o,r)]$ and $\mathbb{E}[p((s, f(o,s)) \mid (r, f(o,r)))]$ using the posterior samples.

Let us consider the same scenario as in Equation (15). Next, we simulate data and study the Kullback-Leibler divergence of the true distribution from the estimated distribution as a function of number of studies and number of reviews. The results are shown in Figure 2.

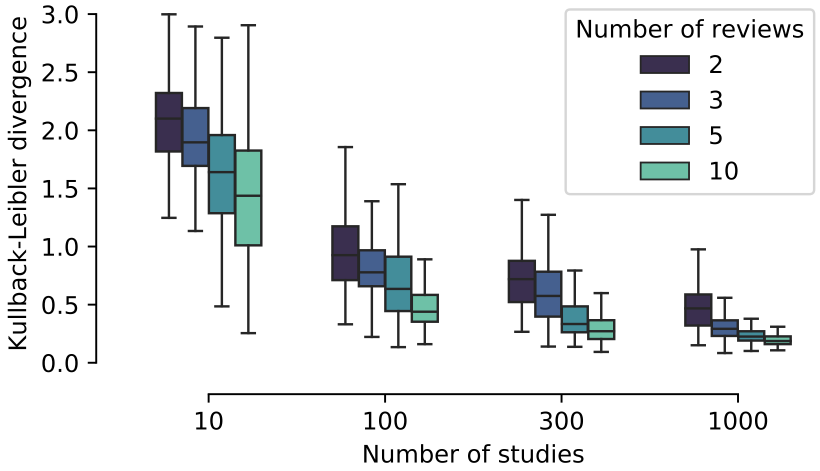

Figure 2: Kullback-Leibler divergence (KLD) is studied as a function of number of studies and number of reviews. Each boxplot is estimated based 100 simulations.

Estimation of error rates

Without a correction for imperfect QA'ing Radiologists

If $N_{\text{reviews}} = 1$ for all $i = 1, 2, \dots, N_{\text{studies}}$, then the maximum likelihood estimates (MLEs) of the rates of discrepancies under the categorical model without a cor-

rection for imperfect QA'ing Radiologists given a severity grade, $g \in \mathcal{G}$, are

$$\begin{aligned}
\hat{p}_{\text{MLE}}(\text{Agree}|g) &= \frac{1}{\sum_{i=1}^{N_{\text{studies}}} [r_i = g]} \sum_{i=1}^{N_{\text{studies}}} [f(o_i, r_i) = \text{Agree} \wedge r_i = g], \\
\hat{p}_{\text{MLE}}(\text{1-degree undercall}|g) &= \frac{1}{\sum_{i=1}^{N_{\text{studies}}} [r_i = g]} \sum_{i=1}^{N_{\text{studies}}} [f(o_i, r_i) = \text{1-degree undercall} \wedge r_i = g], \\
\hat{p}_{\text{MLE}}(\text{1-degree overcall}|g) &= \frac{1}{\sum_{i=1}^{N_{\text{studies}}} [r_i = g]} \sum_{i=1}^{N_{\text{studies}}} [f(o_i, r_i) = \text{1-degree overcall} \wedge r_i = g], \\
\hat{p}_{\text{MLE}}(\text{2-degree undercall}|g) &= \frac{1}{\sum_{i=1}^{N_{\text{studies}}} [r_i = g]} \sum_{i=1}^{N_{\text{studies}}} [f(o_i, r_i) = \text{2-degree undercall} \wedge r_i = g], \\
\hat{p}_{\text{MLE}}(\text{2-degree overcall}|g) &= \frac{1}{\sum_{i=1}^{N_{\text{studies}}} [r_i = g]} \sum_{i=1}^{N_{\text{studies}}} [f(o_i, r_i) = \text{2-degree overcall} \wedge r_i = g],
\end{aligned} \tag{17}$$

where

$$[x] = \begin{cases} 1 & \text{if x is true} \\ 0 & \text{otherwise.} \end{cases} \tag{18}$$

With a correction for imperfect QA'ing Radiologists

If $N_{\text{reviews}} = 1$ for all $i = 1, 2, \ldots, N_{\text{studies}}$, then the maximum likelihood estimates (MLEs) of the rates of discrepancies under the categorical model with a correction for imperfect QA'ing Radiologists given a severity grade, $g \in \mathcal{G}$, are

$$\begin{aligned}
\hat{p}_{\text{MLE}}(\text{Agree}|g) &= \frac{1}{\sum_{i=1}^{N_{\text{studies}}} p\left(g|\left(r_i, f(o_i, r_i)\right)\right)} \sum_{i=1}^{N_{\text{studies}}} p\left((g, \text{Agree}) \mid (r_i, f(o_i, r_i))\right), \\
\hat{p}_{\text{MLE}}(\text{1-degree undercall}|g) &= \frac{1}{\sum_{i=1}^{N_{\text{studies}}} p\left(g|\left(r_i, f(o_i, r_i)\right)\right)} \sum_{i=1}^{N_{\text{studies}}} p\left((g, \text{1-degree undercall}) \mid (r_i, f(o_i, r_i))\right), \\
\hat{p}_{\text{MLE}}(\text{1-degree overcall}|g) &= \frac{1}{\sum_{i=1}^{N_{\text{studies}}} p\left(g|\left(r_i, f(o_i, r_i)\right)\right)} \sum_{i=1}^{N_{\text{studies}}} p\left((g, \text{1-degree overcall}) \mid (r_i, f(o_i, r_i))\right), \\
\hat{p}_{\text{MLE}}(\text{2-degree undercall}|g) &= \frac{1}{\sum_{i=1}^{N_{\text{studies}}} p\left(g|\left(r_i, f(o_i, r_i)\right)\right)} \sum_{i=1}^{N_{\text{studies}}} p\left((g, \text{2-degree undercall}) \mid (r_i, f(o_i, r_i))\right), \\
\hat{p}_{\text{MLE}}(\text{2-degree overcall}|g) &= \frac{1}{\sum_{i=1}^{N_{\text{studies}}} p\left(g|\left(r_i, f(o_i, r_i)\right)\right)} \sum_{i=1}^{N_{\text{studies}}} p\left((g, \text{2-degree overcall}) \mid (r_i, f(o_i, r_i))\right),
\end{aligned} \tag{19}$$

where

$$
\begin{aligned}
p\left(g|\left(r_i, f(o_i, r_i)\right)\right) = & p\left((g, \text{Agree}) \,|\, (r_i, f(o_i, r_i))\right) \\
& + p\left((g, \text{1-degree undercall}) \,|\, (r_i, f(o_i, r_i))\right) \\
& + p\left((g, \text{1-degree overcall}) \,|\, (r_i, f(o_i, r_i))\right) \\
& + p\left((g, \text{2-degree undercall}) \,|\, (r_i, f(o_i, r_i))\right) \\
& + p\left((g, \text{2-degree overcall}) \,|\, (r_i, f(o_i, r_i))\right).
\end{aligned} \tag{20}
$$

Majority

Let us consider the following data

$$
\begin{aligned}
\mathcal{D} = \Big\{ & \left\{ \left(r_{1,1}, f(o_1, r_{1,1})\right), \ldots, \left(r_{1,N_{\text{reviews}}}, f(o_1, r_{1,N_{\text{reviews}}})\right) \right\}, \\
& \left\{ \left(r_{2,1}, f(o_2, r_{2,1})\right), \ldots, \left(r_{2,N_{\text{reviews}}}, f(o_2, r_{2,N_{\text{reviews}}})\right) \right\}, \\
& \vdots \\
& \left\{ \left(r_{N_{\text{studies}},1}, f(o_{N_{\text{studies}}}, r_{N_{\text{studies}},1})\right), \ldots, \left(r_{N_{\text{studies}},N_{\text{reviews}}}, f(o_{N_{\text{studies}}}, r_{N_{\text{studies}},N_{\text{reviews}}})\right) \right\} \Big\}.
\end{aligned} \tag{21}
$$

Then we derive the majority agreement data set by filtering out the studies without majority agreement

$$
\mathcal{D}_{\text{majority}} = \left\{ (y,z) | x \in \mathcal{D} \wedge (y,z) \in x \wedge \sum_{x' \in x} [(y,z) = x'] > \frac{N_{\text{reviews}}}{2} \right\}. \tag{22}
$$

That is, we keep those studies in which there are more than $N_{\text{reviews}}/2$ QA'ing Radiologists that agree with each, moreover, this majority opinion is used as the assessment of the study. In other words, $|\mathcal{D}_{\text{majority}}| \leq |\mathcal{D}|$ and for each study in $\mathcal{D}_{\text{majority}}$ we have a single review instead of having N_{reviews} reviews.

After filtering oout the studies without majority agreement, we use the approach described in Equation (17) to estimate error rates.
